# Erichsen and Humphry

**Published:** 1896-10-03

**Authors:** 


### The Hospital

An Institutional Journal of

The Medical Sciences and Hospital Administration, with

AN EXTRA NURSING SUPPLEMENT.

Vol. XXI.—No. 523.] [Oct. 3, 1896.

### Erichsen and Humphry.

The question most frequently asked concerning the prominent physician or surgeon at his death is, What has he done of an enduring character for the advancement of his science or the improvement of his art? It is a fair and rightly-intentioned question. The two surgeons whose names are placed at the head of this article both died last week—Sir John Erichsen at the comparatively full age of 78, whilst Sir George Murray Humphry was two years younger. Both those surgeons, tried by this eminently modern standard of judging, even dead men, have much to gain and nothing at all to lose.

Sir John Erichsen was a typical surgeon of a generation entirely different in most respects from the typical surgeon of the present day. He had done most of his work before antiseptic surgery was convincingly demonstrated. Much of it he had done even before physiological and pathological laboratories, as we understand them, were thought of. And yet his "Science and Art of Surgery," first published in 1853, and the work of a man who was then no more than thirty-five years of age, is one of the recognised text-books of surgery at the present day, and its latest edition, the tenth, was published so recently as 1895. It would not be possible to furnish a more convincing proof than this of the real personal and professional capacity of Erichsen from the earliest period of his life. For our part we desire, not merely to do something towards keeping green the memory of a very meritorious member of our profession, but our purpose also is to make note of those features in his character and conduct which led to immediate and permanent success and honour, and to point them out for general imitation. Erichsen did nothing extraordinary, but he did certain ordinary things extraordinarily well. That was the secret of secrets in his professional life. If he had not been a careful observer in his earlier years; if he had not been a very thorough and persistent worker; if he had not so used his intelligence as to clearly perceive the full significance of the surgical work he was doing, alike in its great principles and in its details, he could never have written the "Science and Art of Surgery" at the age of thirty-five; and if he had written it, the work could never, in spite of its later transmutations, have survived the manifold surgical developments of the last thirty years, and remained to the present day a standard work. If it be asked what is Erichsen's permanent professional monument, the answer must be, his still vital and still influential "Science and Art of Surgery."

Sir George Murray Humphry, although within two years of Sir John Erichsen in point of age, was a man of a different, and, if we mistake not, a decidedly more modern type. In early life he was a surgeon, but as he grew older he seems to have perceived that medicine and surgery are not two, but one, and in 1859, when nearly forty years of age, he studied for and honourably attained to the degree of M.D. But even with this, and the successive distinctions of the professorships of anatomy and surgery in his own University of Cambridge, he was not satisfied. He was ambitious and enthusiastic, not so much for himself as for the profession to which he belonged, and which he so brilliantly adorned. Like Erichsen, Humphry made the whole profession his debtor, though in a different way. He was not merely a practitioner and a man of science, he was a medical politician, and his political capacity enabled him to restore and build up for his profession a school of medicine at a great English University.

It will never be known how much British medicine has suffered by its exclusion for so many years from our older Universities. To that exclusion was, no doubt, almost entirely due the general disrepute into which the medicine of the last century fell in the estimation of the more cultured classes. When Sir George Humphry commenced his professional career the University of Cambridge possessed a medical school, of which not only was she herself ashamed, but of which the whole profession showed its contempt by carefully avoiding it as a place of education. At this moment the medical school of Cambridge ranks with the highest, and her medical degrees are sought after by many of the most cultured and capable of the young men of the day. The change effected was largely, it may almost be said entirely, due to the enthusiastic, disinterested, and unremitting efforts of Sir George Murray Humphry.

Medicine is not much given to enthusiastic recognitions of its dead leaders. It is, we think, a pity that it should be so. Honourable ideals are of great value; work done, not exclusively for self but for one's profession, deserves not merely a passing recognition, but some gratitude, and that of a real and lasting character. The two surgeons whose work for the profession has been briefly outlined belonged to a class of Englishmen of the very highest type; and, in honouring their memory, it is most of all incumbent upon those of us who are still in the thick of the fight to be endowed with their spirit, and to emulate their loyalty to duty and to the highest interests and honour of our great profession.

